# Integrated Network and Transcriptomic Analyses Identify a Candidate Ethanol–ADAM17 Molecular Axis Associated with Glioblastoma Progression

**DOI:** 10.3390/ijms27177837

**Published:** 2026-09-01

**Authors:** Aakash Arumugam, Hannah Baik, Nayoung Jang, Wenfei Huang, Sulie L. Chang

**Affiliations:** Institute of NeuroImmune Pharmacology, Seton Hall University, South Orange, NJ 07079, USA

**Keywords:** ADAM17, glioblastoma, ingenuity pathway analysis, ectodomain shedding, sheddase signaling, EGFR signaling, inflammatory signaling, tumor microenvironment, TCGA-GBM, extracellular matrix remodeling

## Abstract

Glioblastoma (GBM) is the most aggressive brain tumor, with a median survival of approximately 15 months despite multimodal therapy. Although ethanol (EtOH) exposure has been associated with tumor-promoting inflammatory and signaling alterations, the molecular mediators linking EtOH to GBM progression remain poorly defined. We used Ingenuity Pathway Analysis (IPA) to construct independent EtOH- and ADAM17-associated molecular networks and identify shared regulatory effectors. Comparison of 921 EtOH-associated and 594 ADAM17-associated molecules identified 97 overlapping molecules, of which 25 were retained as high-confidence shared effectors. IPA disease-association analysis identified GBM as the strongest disease association in both the ADAM17 and integrated EtOH–ADAM17 networks. Computational network perturbation analyses indicated that simulated removal of ADAM17 eliminated the GBM disease association, whereas retention of ADAM17 as the central hub produced only a partial association, supporting its role as a key regulator within the predicted network. Analysis of TCGA-GBM transcriptomic data identified significant upregulation of ADAM17, CAV1, FLNA, and CASP8. Canonical pathway analysis further identified enrichment of ADAM17-associated signaling pathways, including sheddase, EGFR, ERBB4, neuregulin, JAK/IL-6, and matrix metalloproteinase signaling. Collectively, these findings support a candidate EtOH–ADAM17 molecular axis associated with GBM progression, providing a rationale for experimental evaluation of ADAM17 as a therapeutic target.

## 1. Introduction

Glioblastoma multiforme (GBM), classified as a WHO grade IV astrocytoma, is the most common and deadliest primary brain tumor in adults [1,2]. Even with the current standard of care, surgical resection followed by concurrent temozolomide chemotherapy and radiation, median survival remains approximately 15 months, and fewer than 10% of patients survive beyond five years [3,4]. Resistance to treatment arises from several converging factors, including marked intratumoral heterogeneity, a highly invasive growth pattern, and the blood–brain barrier, which severely limits drug delivery to the tumor [5,6,7]. Despite sustained research efforts, the molecular events driving GBM initiation and progression remain incompletely understood, highlighting the need to identify new therapeutic targets [8].

Alcohol consumption is a recognized modulator of cancer risk across a wide range of tumor types [9]. Ethanol (EtOH) and its metabolites act on multiple systems by disrupting signal transduction pathways, amplifying inflammatory mediator release, and remodeling the tumor microenvironment in ways that may favor malignant progression [10]. In the context of GBM, Myers et al. (2025) demonstrated that EtOH exposure increases tumor cell migration across the corpus callosum and promotes angiogenesis in a mouse GBM model, providing experimental evidence that alcohol can exacerbate malignant brain tumor biology [11]. Nevertheless, the molecular pathways through which EtOH influences GBM remain incompletely understood.

ADAM17 (A Disintegrin and Metalloproteinase 17), also known as tumor necrosis factor-α converting enzyme (TACE), is a transmembrane metalloprotease that cleaves the extracellular domains of numerous cell-surface proteins, including cytokine receptors, adhesion molecules, and growth factor ligands [12]. By regulating the availability of these proteins at the cell surface, ADAM17 occupies a central position in inflammatory signaling, EGFR transactivation, and tumor invasion pathways, all of which are recognized features of GBM biology [13,14]. Consistent with this role, ADAM17 expression increases with glioma grade and is associated with poor patient outcomes [15]. Mechanistically, Zhang et al. (2018) described a FoxM1–ADAM17–EGFR feed-forward loop that promotes mesenchymal transition in GBM, whereas Yang et al. (2023) demonstrated that ADAM17 contributes to temozolomide resistance and is regulated by miR-145 [16,17,18]. A broader review by Łukaszewicz-Zając et al. (2021) further underscored the importance of ADAM-family proteases, particularly ADAM17, in malignant central nervous system tumors [12]. Through ectodomain shedding of EGFR ligands, cytokines, and adhesion-related molecules, ADAM17 regulates signaling programs involved in inflammation, extracellular matrix remodeling, and tumor invasion, processes that are highly relevant to GBM biology. Because ADAM17 is deeply embedded in both inflammatory biology and cancer progression, two biological processes that ethanol is known to influence, we hypothesized that ADAM17 may serve as a molecular bridge linking EtOH-associated signaling to GBM.

To investigate this hypothesis, we used Ingenuity Pathway Analysis (IPA; QIAGEN Digital Insights, Redwood City, CA, USA), a curated bioinformatics platform that constructs molecular interaction networks from published literature curated in the QIAGEN Knowledge Base (QKB) [19]. We generated independent EtOH- and ADAM17-centered networks, identified their shared molecular components, and evaluated whether these shared molecules preferentially converge on GBM-related biology. Because IPA integrates previously reported molecular relationships rather than directly measuring molecular interactions, the resulting networks are interpreted as hypothesis-generating models that require experimental validation.

We then performed a secondary analysis of transcriptomic data from The Cancer Genome Atlas glioblastoma (TCGA-GBM) cohort to determine whether candidate molecules identified through network analysis exhibited altered expression in patient tumors and to characterize associated signaling pathways by pathway enrichment analysis. Thus, IPA network modeling generated mechanistic hypotheses, whereas TCGA transcriptomic analysis provided independent observational evidence from patient-derived data. By integrating hypothesis-generating network analysis with patient-derived transcriptomic data, this study reveals a potential EtOH–ADAM17 molecular axis associated with GBM progression and provides a framework for identifying candidate molecular mechanisms for future experimental investigation.

## 2. Results

### 2.1. Overlap Between EtOH- and ADAM17-Associated Molecular Networks

**Figure 1:**
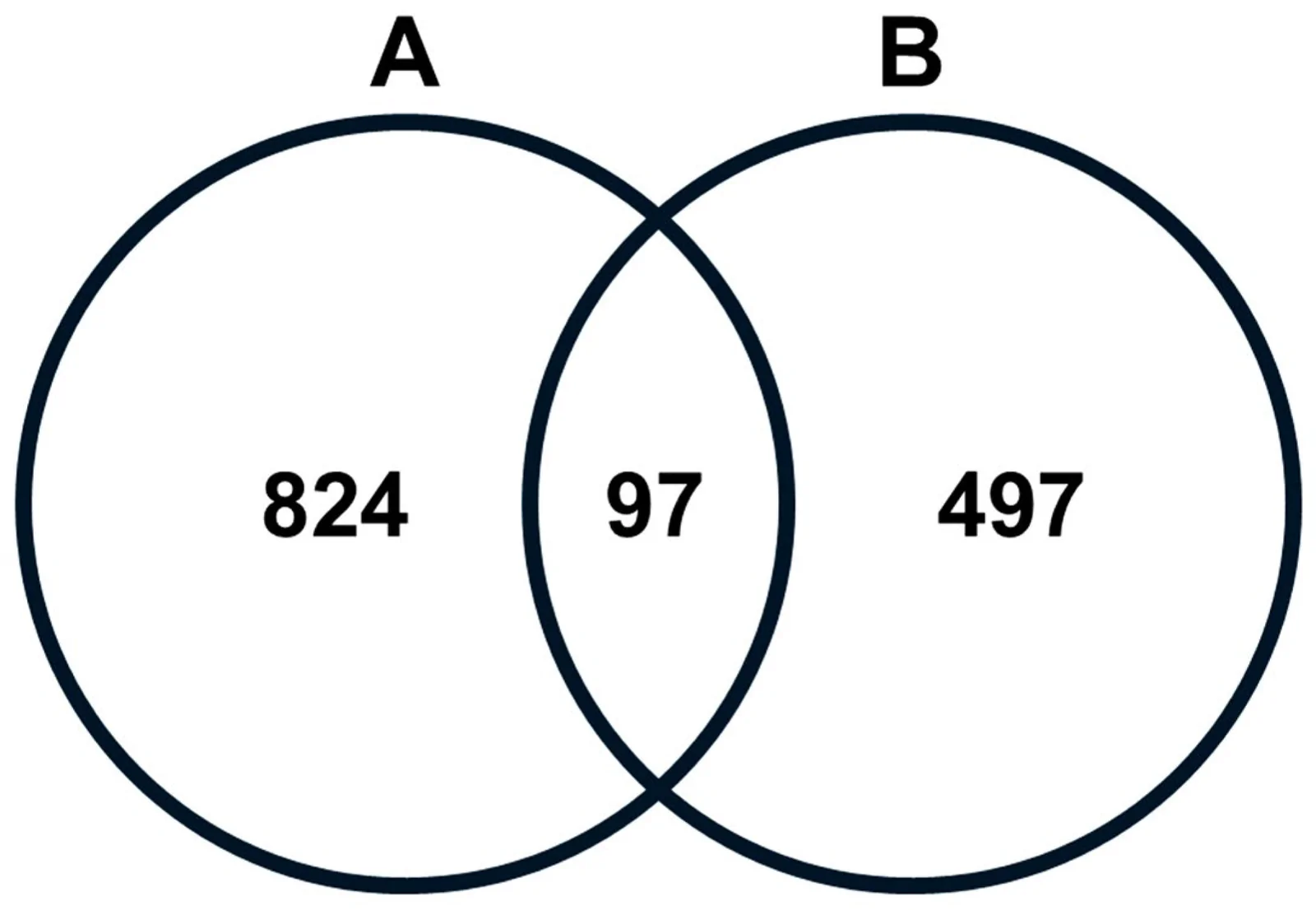
Venn diagram of EtOH- and ADAM17-associated molecular networks. This diagram illustrates the overlap between IPA-generated molecular networks comprising 921 EtOH-associated molecules (List A) and 594 ADAM17-associated molecules (List B), retrieved from the QIAGEN Knowledge Base. Ninety-seven molecules were shared between the two networks.

To assess shared molecular components between ethanol (EtOH) signaling and ADAM17-associated activity, two independent IPA networks were generated from molecules retrieved from the QIAGEN Knowledge Base (QKB). The EtOH-associated network comprised 921 molecules, and the ADAM17-associated network comprised 594 molecules. Comparison using IPA’s Compare function identified 97 shared molecules between the two networks (Figure 1, Tables S1 and S2). This overlap corresponds to approximately 10.5% of the EtOH network and 16.3% of the ADAM17 network.

### 2.2. Identification of 25 Putative ADAM17-Mediated Intermediates of EtOH Signaling

**Figure 2:**
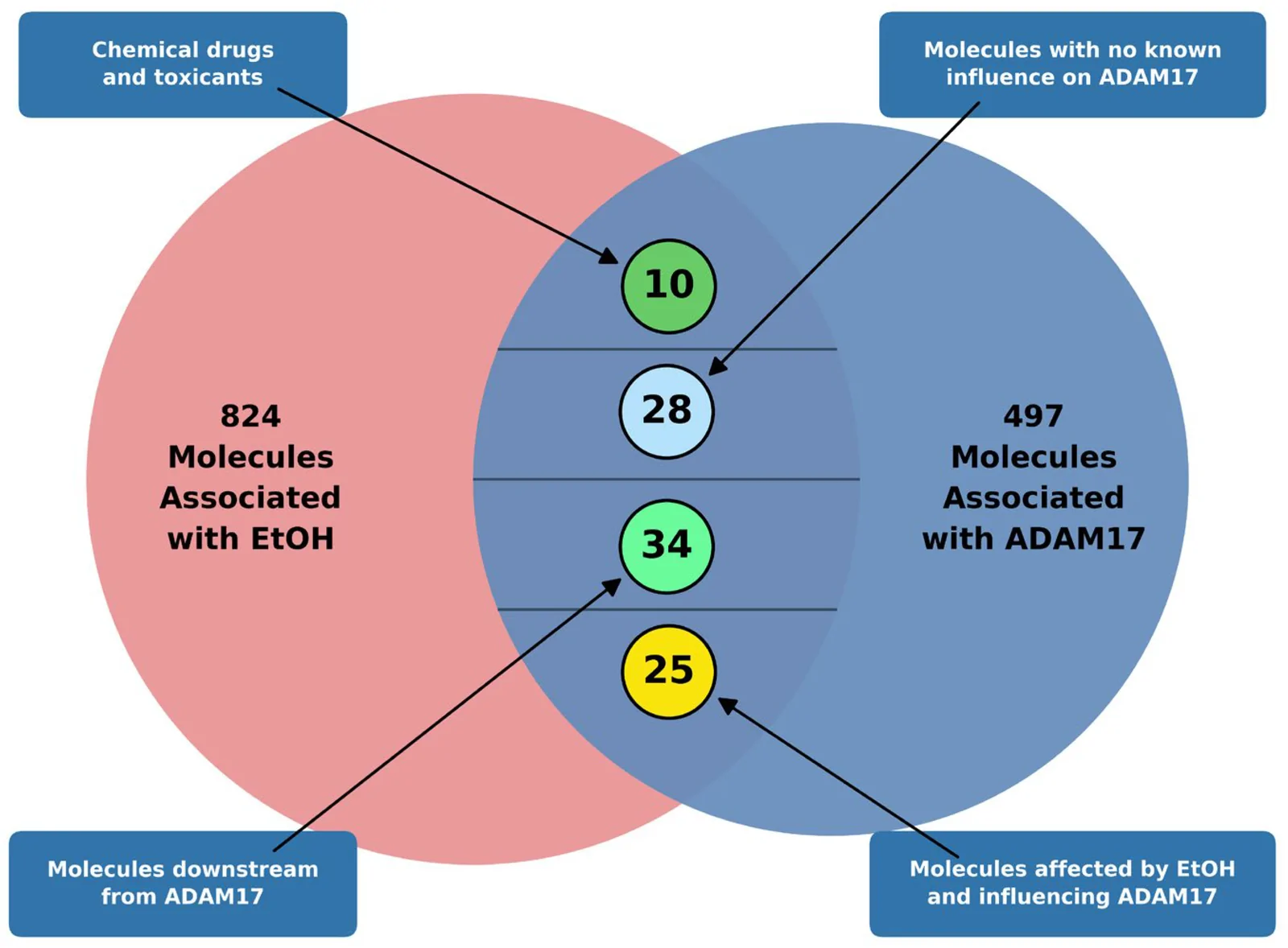
Categorization of the EtOH–ADAM17 molecular overlap. Venn diagram showing categorization of 97 overlapping molecules between the EtOH- and ADAM17-associated networks. Among these 97 molecules, 10 were chemical drugs or toxicants, 28 lacked directional association with ADAM17, and 34 were identified as downstream ADAM17 effectors. The remaining 25 molecules (highlighted in yellow) were retained as EtOH downstream molecules positioned upstream of ADAM17, representing putative intermediates linking EtOH signaling to ADAM17 activity.

Of the 97 overlapping molecules, 10 were categorized as chemical drugs or toxicants and were excluded as non-biological intermediates, leaving 87 molecules for further analysis. The directional relationship between EtOH and ADAM17 was then evaluated using IPA’s Path Explorer tool, with EtOH designated as the origin node (Set A) and ADAM17 designated as the terminal node (Set B); only paths in the EtOH-to-ADAM17 direction were queried. This directional Path Explorer query identified 53 molecules situated along a biological path connecting EtOH to ADAM17, each displaying a colored (orange or blue) connecting edge from EtOH, indicating a confirmed, direction-specific causal effect of EtOH on the intermediate molecule. The remaining 34 molecules were not returned by this forward-directional query; rather, these molecules were identified as downstream effectors of ADAM17, meaning their documented relationship to ADAM17 runs in the reverse direction (from ADAM17 toward the molecule) rather than from EtOH toward ADAM17, and were therefore excluded as they do not represent EtOH-associated intermediates positioned upstream of ADAM17. Each of the 53 EtOH-linked molecules was further evaluated based on the nature of its connecting relationship to ADAM17 as visually and algorithmically represented within IPA. Twenty-five molecules displayed colored directional edges to ADAM17 (orange, denoting predicted activation of ADAM17; or blue, denoting predicted inhibition of ADAM17), indicating a continuous, consistent causal path from EtOH through the intermediate to ADAM17. The remaining 28 molecules, while linked to EtOH by a colored edge, displayed an uncolored (grey) connecting edge specifically to ADAM17, indicating that IPA could not generate a directional prediction for this relationship; this reflects an absence of traceable causal evidence linking the intermediate to ADAM17 within the QKB, such that the pathway signal from EtOH could not be shown to propagate through the intermediate to ADAM17 specifically. Molecules displaying this uncolored state relative to ADAM17 were excluded from further analysis. The remaining 25 molecules, all displaying colored, direction-specific connecting edges to ADAM17, were retained as putative intermediates linking EtOH signaling to ADAM17-associated activity (Figure 2, Table S3). These molecules, including key nodes such as CAV1, FLNA, CASP8, CXCL12, ESR1, TNF, and miR-145, were carried forward as the core molecular set for downstream disease association analysis.

### 2.3. IPA Disease Association Analysis Identifies Glioblastoma as the Primary Disease

**Figure 3:**
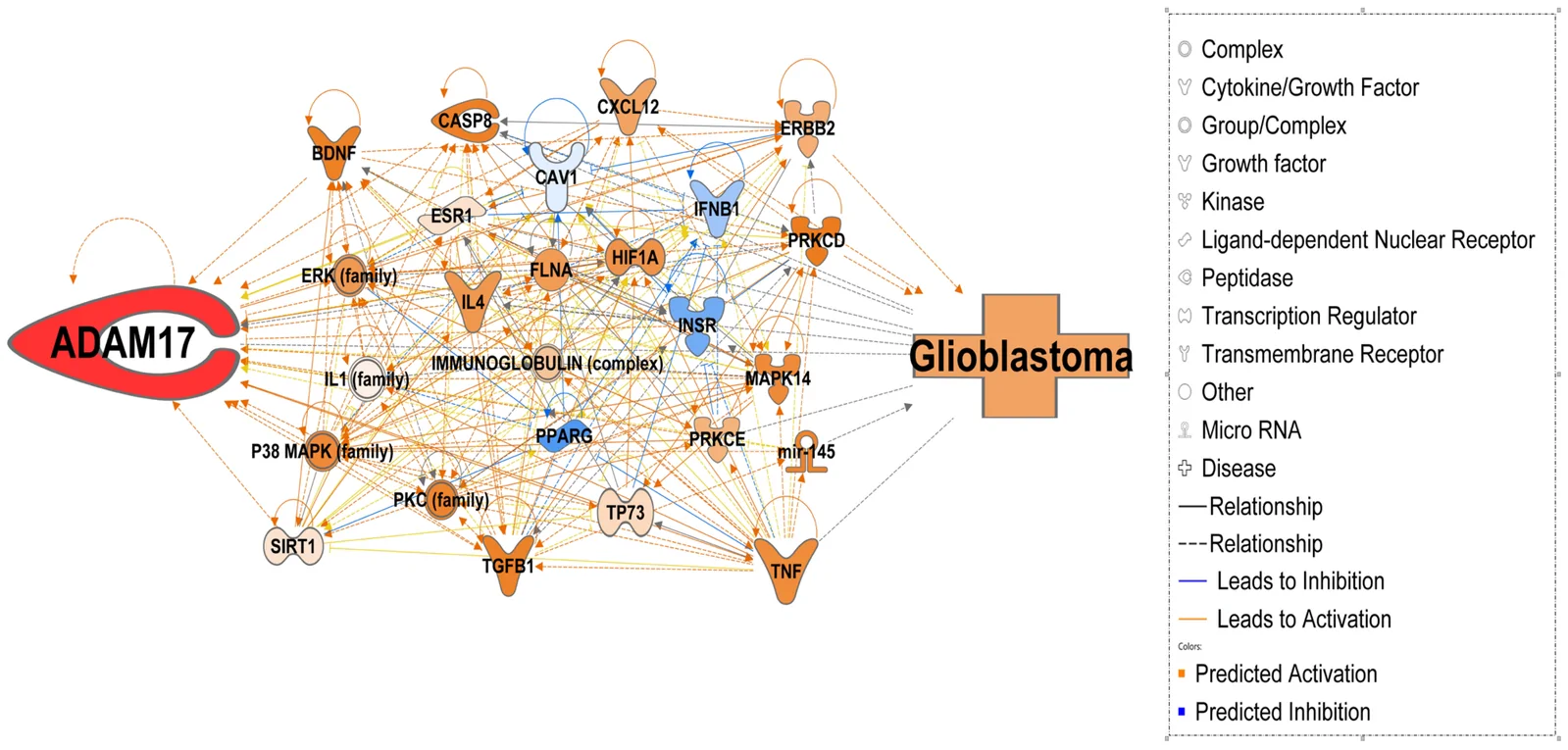
ADAM17–glioblastoma network mediated by 25 intermediate molecules. IPA network diagram illustrating the relationship between ADAM17 (left, red diamond) and glioblastoma (right, orange rectangle) through 25 intermediate molecules identified from the EtOH–ADAM17 overlap analysis. The network shows a significant disease association (*p* = 4.03 × 10^−13^). Legend shown on right is consistent with descriptions for Figures 4, 5 and 7–9.

The 25 putative intermediate molecules linking EtOH signaling to ADAM17 activity were analyzed using the IPA Grow-Disease and Functions function to identify disease associations. Within the ADAM17-associated network, glioblastoma emerged as a significantly associated disease (*p* = 4.03 × 10^−13^; Figure 3, Scheme S1, Raw File S1). This highly significant enrichment indicates that the identified intermediate molecules are strongly associated with known glioblastoma-related biological processes and supports a potential role for ADAM17-mediated signaling in the molecular response to EtOH.

### 2.4. Integration of the EtOH and ADAM17 Networks Strengthens the Association with Glioblastoma

**Figure 4:**
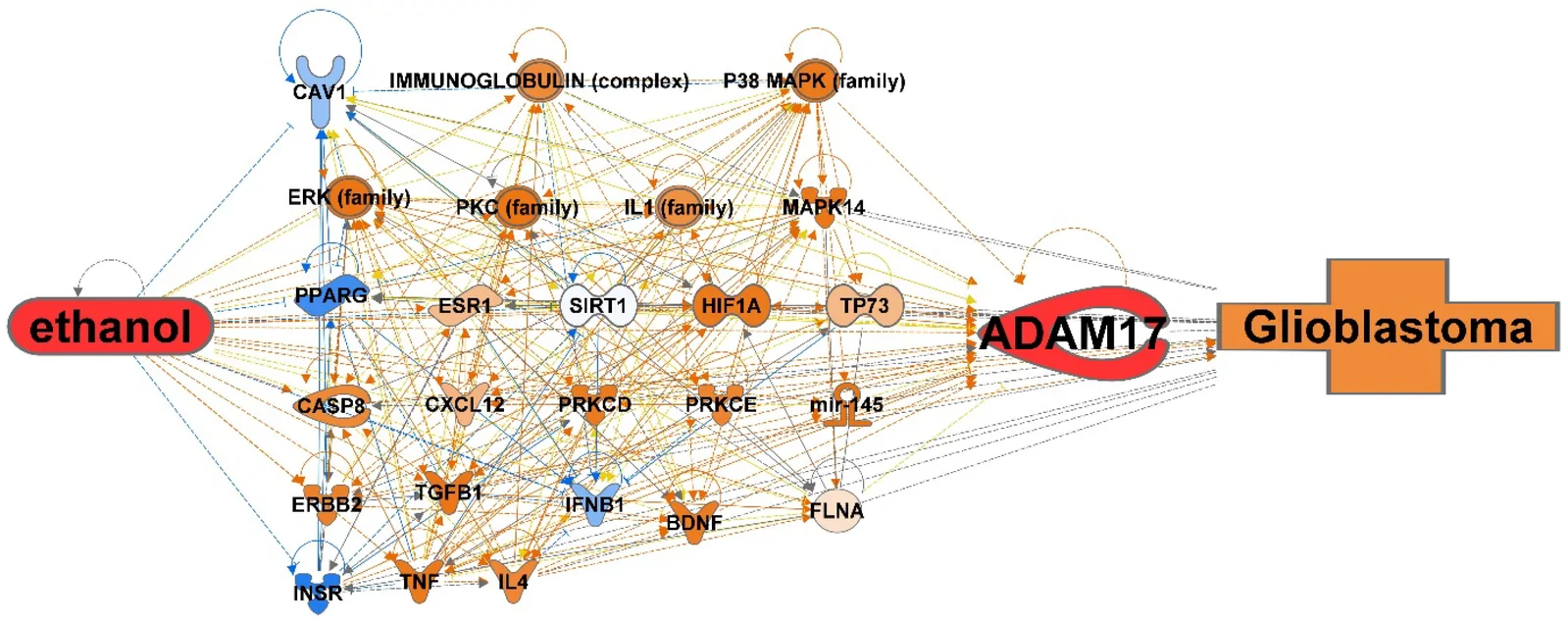
Integrated EtOH–ADAM17–glioblastoma signaling network. IPA network diagram illustrating the integrated signaling network linking ethanol (EtOH; left) to glioblastoma (far right) through ADAM17 (right of center, red) and 11 directly bridging molecules identified from the integrated network analysis. The combined network exhibited a highly significant association with glioblastoma (*p* = 8.59 × 10^−13^), supporting a model in which ADAM17 functions as a putative molecular mediator of EtOH-associated glioblastoma signaling. Please refer to the legend included in Figure 3.

Integration of the EtOH-associated network with the ADAM17-associated network generated an IPA signaling network linking EtOH to glioblastoma through ADAM17. Of the 25 effector molecules identified in Section 2.2, 10 directly connected EtOH to the glioblastoma disease node through ADAM17; together with ADAM17 itself, these constitute the 11-molecule bridging set reported below, while the remaining 15 effector molecules did not participate in this direct connection. Within the integrated network, 11 molecules directly connected EtOH to the glioblastoma disease node through ADAM17. Disease and Functions analysis of the integrated network identified glioblastoma as a significantly associated disease with a high level of significance (*p* = 8.59 × 10^−13^; Figure 4, Scheme S2, Raw File S2).

**Figure 5:**
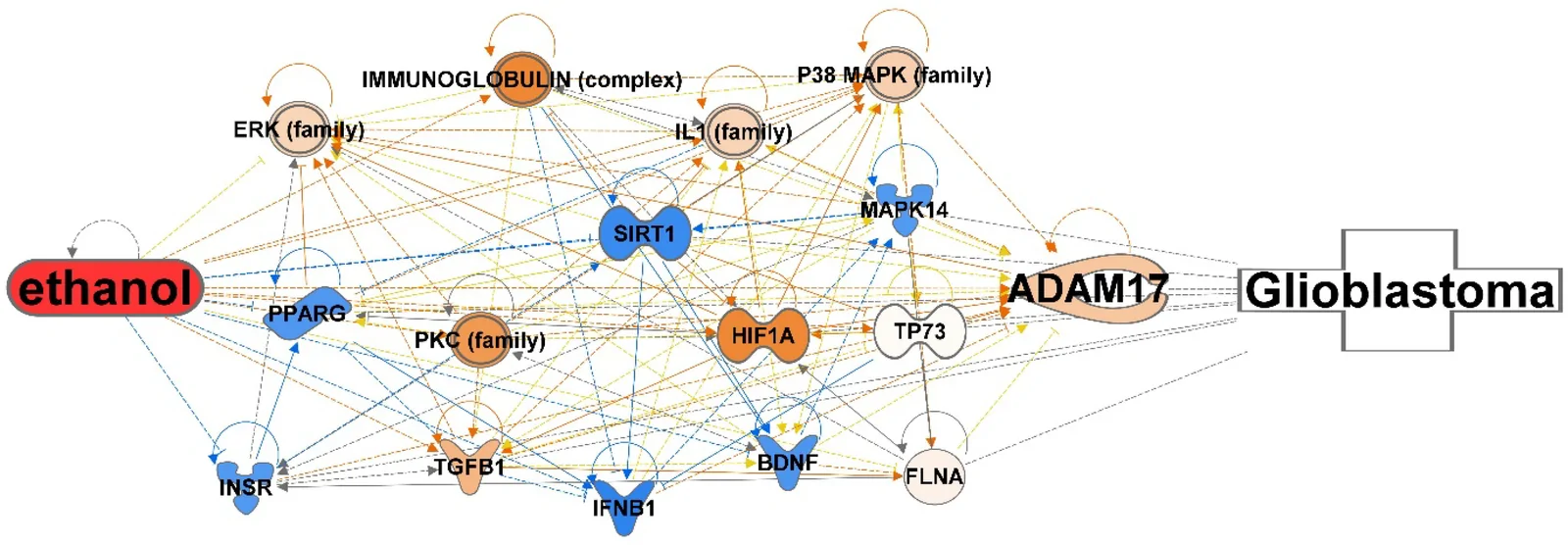
Molecular specificity control of the integrated EtOH–ADAM17–glioblastoma network. IPA network diagram showing the molecular specificity control in which EtOH is connected to 15 non-bridging molecules while ADAM17 remains within the network. Under these conditions, no significant association with glioblastoma was identified by IPA Disease and Function analysis. In contrast to the integrated network shown in Figure 4, replacement of the 11 bridging molecules with the 15 non-bridging molecules abolished the significant glioblastoma association. Please refer to the legend included in Figure 3.

A key question is whether the significant glioblastoma association observed in the integrated network (Figure 4) reflects a specific contribution of the 11 bridging molecules, or whether it could arise from nonspecific features of the network, such as its overall size or the mere presence of ADAM17 and EtOH as network anchors. To distinguish between these possibilities, a molecular specificity control analysis was performed in which the 11 bridging molecules were replaced with 15 non-bridging molecules drawn from the broader EtOH-associated network, while ADAM17 and EtOH were both retained as network anchors and overall network size was preserved. Under these substituted conditions, IPA Disease and Functions analysis did not identify a significant association with glioblastoma (Figure 5, Scheme S3, Raw File S3). Because ADAM17, EtOH, and comparable network size were held constant between the original and control networks, this loss of significance indicates that the glioblastoma association is not attributable to these nonspecific network features, and instead depends specifically on the identity of the 11 bridging molecules.

### 2.5. TCGA-GBM Validation Confirms Upregulation of Key Network Molecules

**Figure 6:**
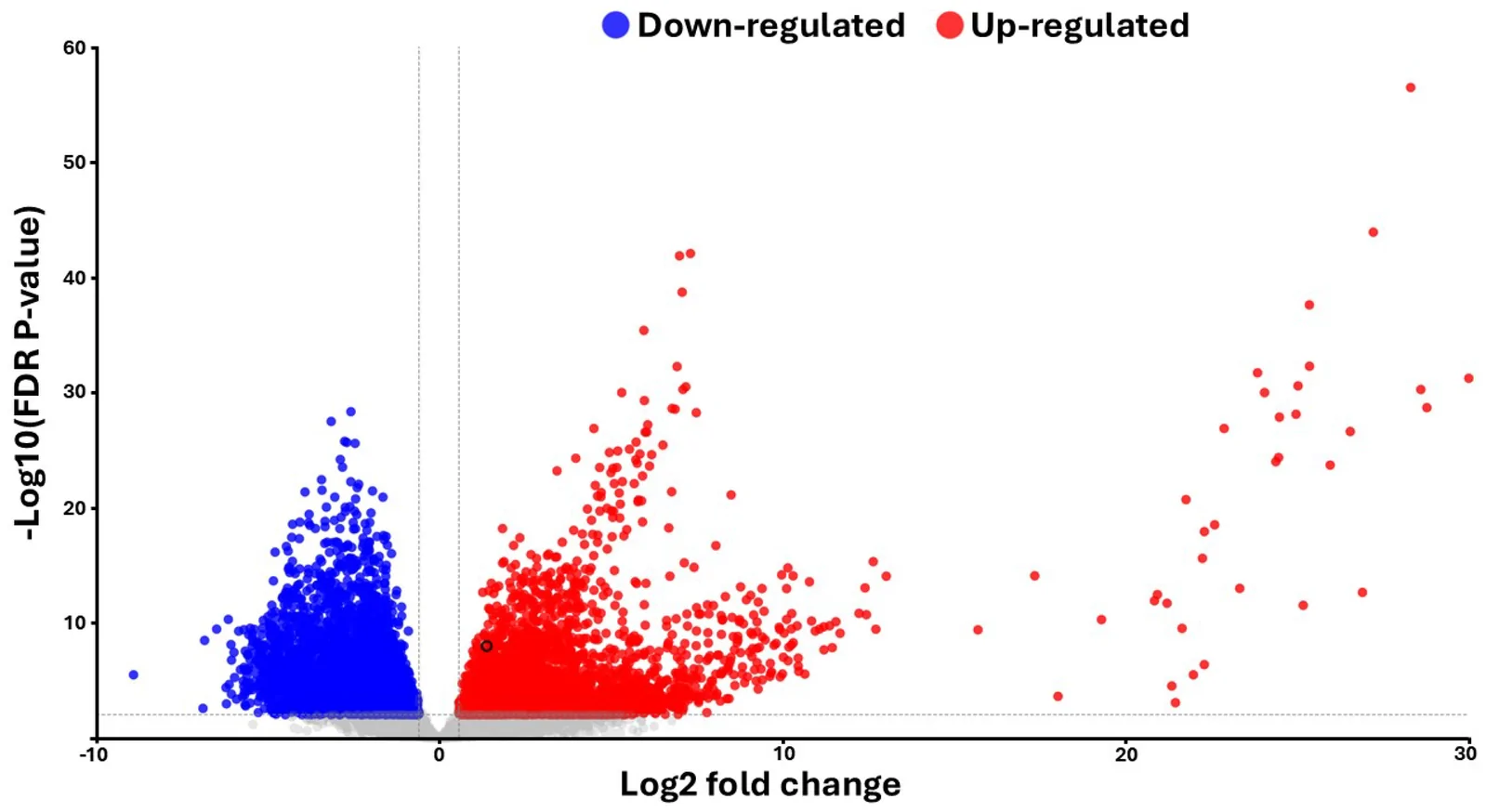
Differential gene expression analysis in TCGA-GBM reveals upregulation of key network molecules. Volcano plot showing differential gene expression in TCGA glioblastoma (GBM) samples (Log2 fold change vs. −Log10 FDR). Red points indicate significantly upregulated genes, and blue points indicate significantly downregulated genes. Key network-associated molecules, including ADAM17, CAV1, FLNA, and CASP8, are highlighted and fall within the upregulated quadrant, supporting their increased expression in GBM.

To validate whether the IPA-predicted molecular axis in human glioblastoma, we examined the expression of the 11 bridging molecules in the TCGA-GBM dataset. Of these, six genes showed statistically significant differential expression (FDR < 0.01 and |log2 fold change| ≥ 1) relative to normal brain tissue (Figure 6). Four molecules were significantly upregulated in GBM, including ADAM17 (log2FC = +1.388; FDR = 1.06 × 10^−8^), CAV1 (log2FC = +2.676; FDR = 9.02 × 10^−5^), FLNA (log2FC = +3.094; FDR = 5.32 × 10^−10^), and CASP8 (log2FC = +2.228; FDR = 3.62 × 10^−10^). Two molecules were significantly downregulated, including PRKCD (log2FC = −1.913) and PRKCE (log2FC = −3.321). The remaining molecules—CXCL12, ESR1, IL4, TNF, and miR-145—were not statistically significantly changed in TCGA-GBM patients. Collectively, these findings demonstrate that a subset of the IPA-identified bridging molecules is differentially expressed in GBM, supporting their potential involvement in the tumor-associated molecular network. Having established that multiple bridging molecules are differentially expressed in human GBM, we next examined whether ADAM17 functions as the central organizational node of the predicted EtOH–ADAM17–GBM network through a series of computational perturbation analyses.

### 2.6. Computational Simulation of ADAM17 Inhibition Reduces GBM Disease Association Despite an Intact Network

**Figure 7:**
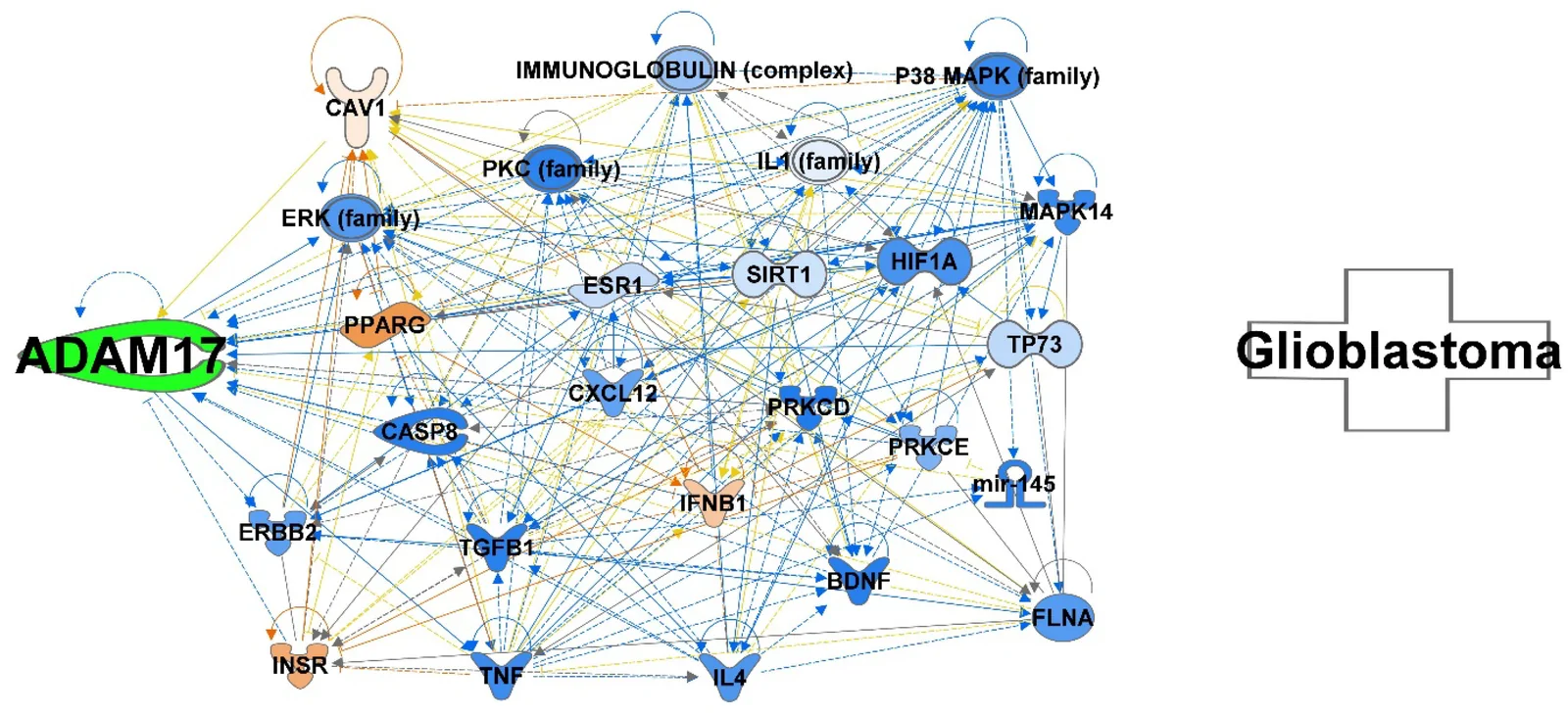
IPA network analysis of computational simulation of ADAM17 inhibition. Ingenuity Pathway Analysis (IPA) network showing computational simulation of ADAM17 inhibition with all 25 effector molecules retained. The glioblastoma (GBM) node (right) does not show increased disease association under simulated ADAM17 inhibition, while the full co-effector network structure remains intact.

To determine whether ADAM17 is required for glioblastoma-associated signaling or functions as one of multiple contributing nodes, computational simulation of ADAM17 inhibition (conducted using IPA’s Molecule Activity Predictor (MAP) tool) was performed within the 25-molecule network while all co-effector molecules were retained. Under these conditions, the glioblastoma (GBM) node demonstrated no association with the molecular network (Figure 7, Scheme S4, Raw File S4), despite preservation of the broader network structure.

### 2.7. ADAM17 Hub Configuration Without Lateral Connectivity Produces Partial GBM Association

**Figure 8:**
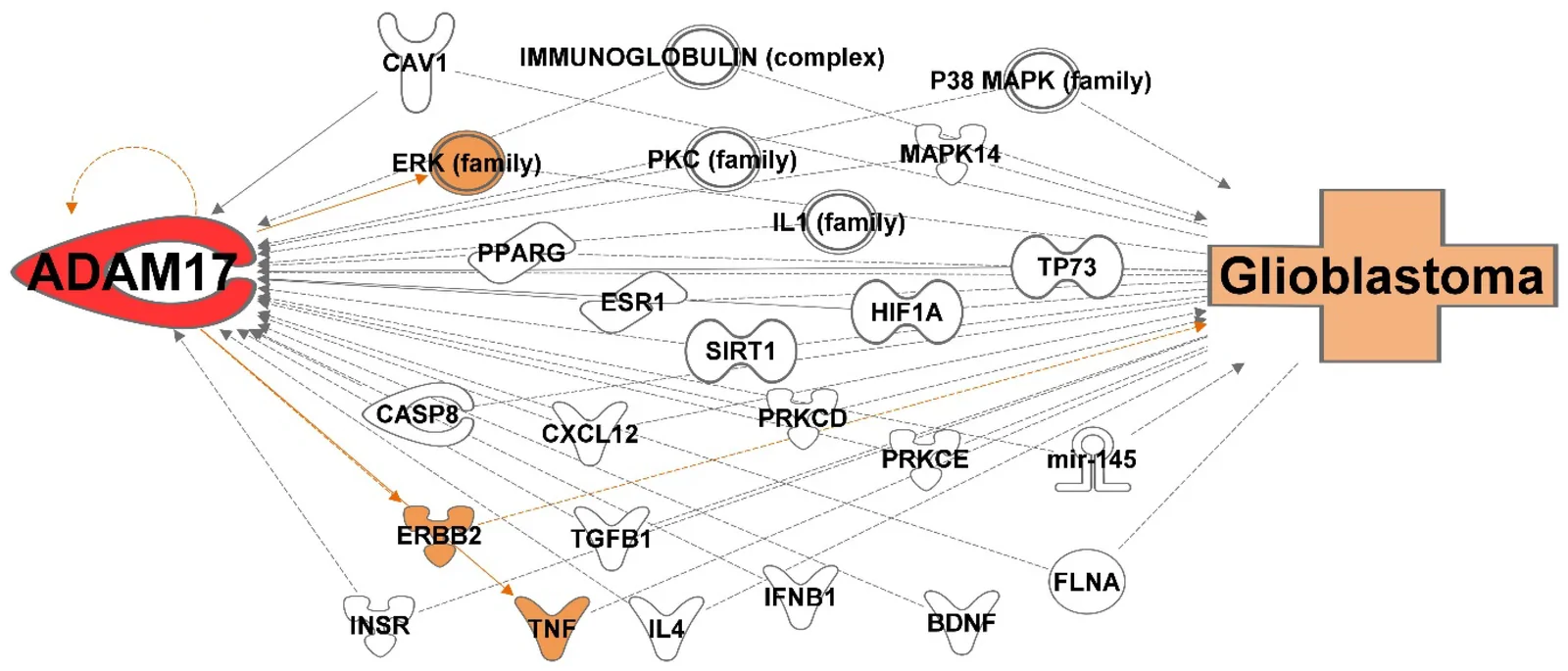
IPA network analysis with ADAM17 as the sole hub following removal of lateral inter-molecular connections. Ingenuity Pathway Analysis (IPA) network showing ADAM17 retained as the central hub with lateral inter-molecular connections removed. The glioblastoma (GBM) association is increased relative to the simulated ADAM17 inhibition condition (Figure 7) but remains lower than in the fully interconnected network (*p* = 2.73 × 10^−11^). Please refer to the legend included in Figure 3.

To distinguish the contribution of ADAM17 from that of broader network connectivity, a computational simulation was performed in which ADAM17 was retained as the central hub while lateral inter-molecular connections among the 25 effector molecules were removed, thereby restricting signaling flow through ADAM17-mediated connectivity. This hub-only configuration resulted in a partial increase in glioblastoma (GBM) disease association, which was greater than the simulated ADAM17 inhibition condition (Section 2.6) but lower than that observed in the fully interconnected network (Figure 3 and Figure 8, Scheme S5, Raw File S5).

### 2.8. Removal of ADAM17 from the EtOH-Stimulated Network Eliminates GBM Disease Association

**Figure 9:**
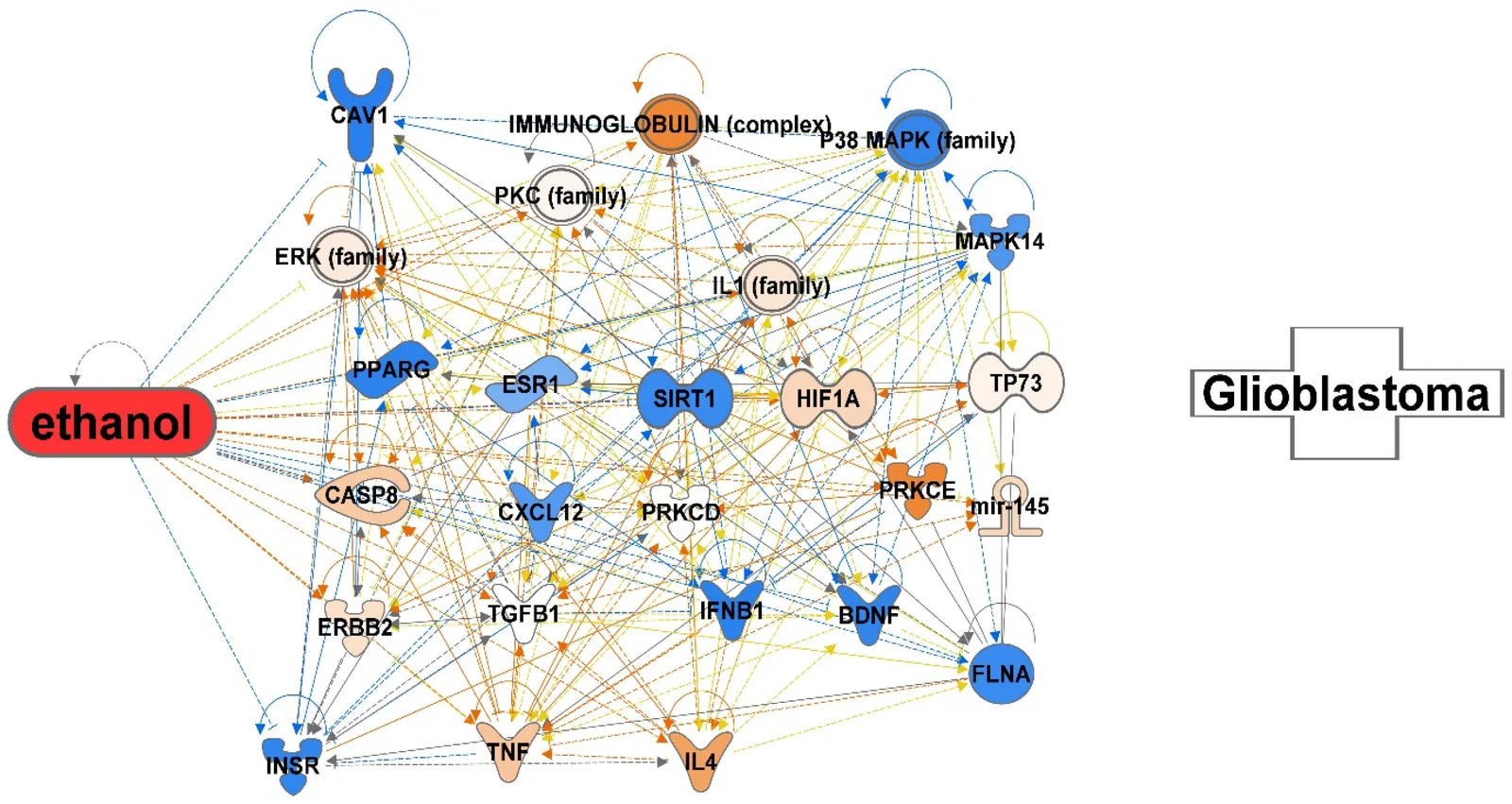
IPA network analysis of the 25-molecule EtOH-stimulated network following removal of ADAM17. Ingenuity Pathway Analysis (IPA) network showing the EtOH-stimulated 25-molecule network with ADAM17 removed. EtOH (left, red) occupies the central hub position; however, the glioblastoma (GBM) node does not show a significant increase in disease association under this condition. Please refer to the legend included in Figure 3.

To further evaluate the role of ADAM17 within the EtOH-stimulated network, a computational simulation was performed in which ADAM17 was removed from the 25-molecule network while EtOH was retained as the central hub. Under these conditions, the remaining effector molecules remained connected to the EtOH-centered network; however, the glioblastoma (GBM) node showed no significant increase in disease association (Figure 9, Scheme S6, Raw File S6).

### 2.9. Upstream Regulator Analysis Identifies ADAM17-Associated Signaling Molecules in TCGA-GBM

To further examine regulatory mechanisms associated with the TCGA-GBM expression profile, IPA Upstream Regulator Analysis (URA) was performed on the TCGA-GBM dataset. Although ADAM17 itself was not identified as a predicted upstream regulator, several established ADAM17 substrates and downstream signaling mediators were among the most significantly activated regulators, including TNF, IL-6, ERBB2, EGFR, and TGFA (Table 1), all of which exceeded the |z-score| ≥ 2 significance threshold applied in this analysis. This pattern is consistent with activation of ADAM17-associated signaling pathways in GBM and provides independent support for the ADAM17-centered molecular network identified through computational analyses.

### 2.10. TCGA-GBM Core Analysis Identifies Enrichment of ADAM17-Related Canonical Pathways

**Figure 10:**
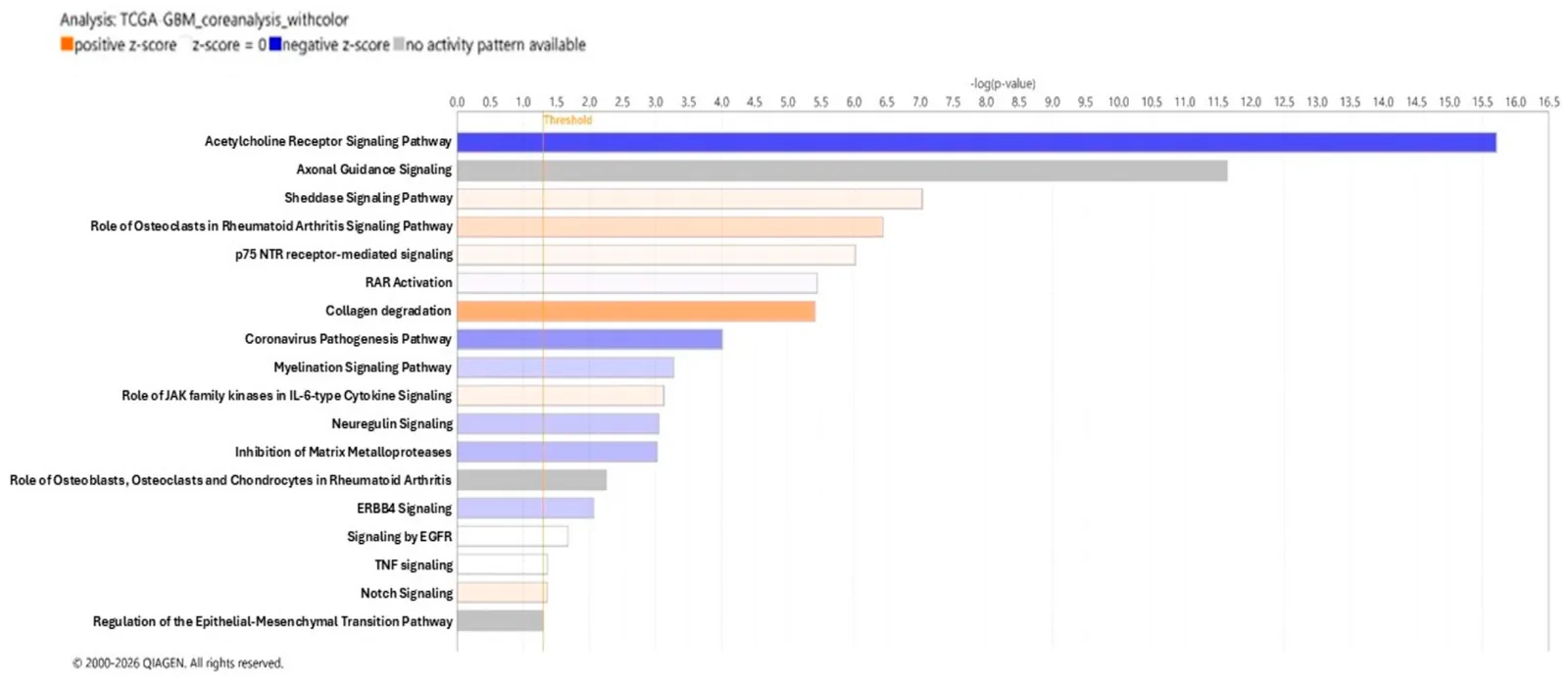
IPA Core Analysis identifies enrichment of canonical signaling pathways in the TCGA-GBM differential expression dataset. Ingenuity Pathway Analysis (IPA) Core Analysis showing canonical pathway enrichment based on differential gene expression in the TCGA glioblastoma (GBM) dataset. The x-axis represents −log(*p*-value). Orange bars indicate pathways with positive z-scores (predicted activation), whereas blue bars indicate pathways with negative z-scores (predicted inhibition). Canonical pathways shown include the Sheddase Signaling Pathway, ERBB4 Signaling, Signaling by EGFR, Neuregulin Signaling, Role of JAK Family Kinases in IL-6-type Cytokine Signaling, Inhibition of Matrix Metalloproteinases, and other significantly enriched pathways identified in the TCGA-GBM dataset. Figure generated using IPA in accordance with QIAGEN’s citation guidelines (https://digitalinsights.qiagen.com/citation-guidelines/ accessed on 13 August 2026). © 2000–2026 QIAGEN. All rights reserved.

To further evaluate the biological relevance of the computationally identified ADAM17-centered network, the TCGA-GBM differential gene expression dataset was analyzed using the IPA Core Analysis workflow, with results filtered to molecules known to interact with ADAM17 within the QKB. Multiple canonical pathways were significantly enriched within this ADAM17-interactor-filtered dataset (Figure 10, Scheme S7, Raw File S7). The Acetylcholine Receptor Signaling Pathway ranked as the most significantly enriched pathway overall (94 molecules; z = −4.904; *p* = 1.93 × 10^−16^) [20]. Additional significantly enriched pathways with established mechanistic ties to ADAM17 signaling included the Sheddase Signaling Pathway (76 molecules; z = 0.688; *p* = 9.34 × 10^−8^), Collagen Degradation (30 molecules; z = 4.747; *p* = 3.91 × 10^−4^), Signaling by EGFR (18 molecules; *p* = 2.1 × 10^−2^), Role of JAK Family Kinases in IL-6-type Cytokine Signaling (30 molecules; z = 0.784; *p* = 7.53 × 10^−4^), ERBB4 Signaling (23 molecules; z = −1.528; *p* = 8.62 × 10^−3^), Neuregulin Signaling (40 molecules; z = −1.633; *p* = 8.93 × 10^−4^), and Inhibition of Matrix Metalloproteinases (17 molecules; z = −1.941; *p* = 9.39 × 10^−4^) [20].

### 2.11. Supplementary Validation Using an Independent Normal-Tissue Reference Dataset

To further evaluate the robustness of the differential expression findings given the limited number of normal brain samples available in the TCGA-GBM cohort (n = 5), an independent supplementary analysis was performed using GEPIA3 [21], which integrates TCGA and GTEx transcriptomic data. Using the limma method with thresholds of |log2FC| ≥ 1 and q ≤ 0.05, 166 TCGA-GBM tumor samples were compared against 110 normal brain samples from GTEx. Five of the six key network molecules identified in the original TCGA-GBM analysis (ADAM17, CAV1, FLNA, CASP8, and PRKCE) were also significantly differentially expressed in this independent, more balanced analysis; PRKCD, which was significant in the original analysis, did not reach significance in the GEPIA3 comparison.

## 3. Discussion

The present study was designed as a stepwise systems biology investigation integrating knowledge-based network analysis with patient-derived transcriptomic analysis to examine the potential mechanistic relationship between ethanol (EtOH) exposure, ADAM17 signaling, and glioblastoma. The analyses integrated a literature-derived knowledge base and patient-derived transcriptomic data through four sequential stages. First, knowledge discovery using the QKB identified a candidate molecular bridge connecting EtOH-associated molecular signatures with ADAM17-related signaling networks [19]. Second, disease association analysis indicated that this molecular bridge was preferentially associated with glioblastoma. Third, computational perturbation analyses identified ADAM17 as a central organizing node within the predicted signaling network. Finally, independent analyses of the TCGA-GBM dataset, including differential gene expression analysis, Upstream Regulator Analysis (URA) [19], and Canonical Pathway Analysis (CPA) [20], provided transcriptomic evidence consistent with the proposed ADAM17-centered molecular network in human glioblastoma. Collectively, these findings support a mechanistic framework linking EtOH-associated molecular signaling to glioblastoma through ADAM17-mediated network interactions.

The QIAGEN Knowledge Base (QKB) is a manually curated knowledge repository that integrates molecular entities (e.g., genes, proteins, chemicals, and biological processes) and their experimentally supported relationships extracted from the published biomedical literature [19]. Unlike transcriptomic or other experimental datasets, the QKB does not contain primary biological measurements. Instead, it serves as a curated resource that enables computational inference of molecular relationships based on experimentally supported findings reported in the biomedical literature. In this study, Ingenuity Pathway Analysis (IPA) was applied to the QKB to identify literature-supported molecular networks and infer signaling relationships potentially connecting EtOH exposure with ADAM17-associated pathways and glioblastoma biology.

In contrast, the TCGA-GBM dataset consists of primary transcriptomic (RNA-sequencing) data generated from glioblastoma patient tumors and normal brain tissues [22]. Differentially expressed genes (DEGs) identified from the TCGA-GBM cohort were subsequently analyzed using Ingenuity Pathway Analysis (IPA), including Upstream Regulator Analysis (URA) and Canonical Pathway Analysis (CPA), to characterize upstream regulatory mechanisms and biological pathways associated with patient-derived gene expression changes [19]. Thus, whereas QKB-based IPA analysis generated mechanistic hypotheses based on curated experimental knowledge from the published literature, the TCGA-GBM analysis provided orthogonal, patient-derived observational evidence supporting the relevance of these literature-based predictions. The integration of a curated, literature-derived knowledge base with an independent patient transcriptomic dataset represents a complementary systems biology strategy that combines knowledge-driven hypothesis generation with patient-derived evidence. By integrating these complementary sources of evidence, our study provides a comprehensive mechanistic framework supporting the proposed EtOH–ADAM17–glioblastoma signaling axis.

ADAM17 is a membrane-bound metalloprotease that functions primarily as a post-translational sheddase, catalyzing the proteolytic cleavage of the extracellular domains of numerous membrane-bound cytokines, growth factors, growth factor receptors, and adhesion molecules [23,24,25,26]. Through this ectodomain shedding activity, ADAM17 regulates the bioavailability and signaling of multiple pathways involved in inflammation, cell proliferation, migration, and tumor progression [23,27]. Unlike transcription factors or other gene regulatory proteins, ADAM17 exerts its biological effects through rapid and reversible post-translational regulation of substrate accessibility to its catalytic domain rather than through direct regulation of gene transcription [28]. Previous studies have further demonstrated that ADAM17-mediated substrate shedding is dysregulated in glioblastoma, implicating ADAM17 as an important mediator of GBM-associated signaling [16,29]. This established biology provides the mechanistic framework for interpreting the computational findings of the present study.

The principal finding derived from the QKB analysis is that EtOH and ADAM17 share a molecular interaction network whose intersection is significantly enriched for GBM-associated biology, a computational prediction that was subsequently supported by analyses of TCGA GBM patient tumors. Comparison of 921 EtOH-associated molecules with 594 ADAM17-associated molecules identified 97 shared molecules, of which 25 satisfied stringent dual-pathway filtering criteria requiring documented involvement in both EtOH- and ADAM17-associated signaling (Figure 1 and Figure 2). Disease and Functions analysis identified glioblastoma as the most significantly associated disease for this molecular set, and integration of the EtOH and ADAM17 networks further strengthened this association (Figure 3, Figure 4 and Figure 5). Importantly, molecular specificity controls eliminated the statistically significant GBM association, indicating that the observed enrichment depended on the biologically relevant bridging molecules rather than simply on network size or EtOH simulation (Figure 5). Together, these findings implicate a plausible molecular framework through which EtOH-associated signaling may converge on ADAM17-mediated pathways to influence glioblastoma biology.

The biological relevance of this computational network was supported by independent analyses of the TCGA-GBM dataset (Figure 6). Four of the predicted bridging molecules, ADAM17, CAV1, FLNA, and CASP8, were significantly upregulated in patient tumors, whereas PRKCD and PRKCE were significantly downregulated. CAV1 and FLNA have established roles in receptor tyrosine kinase signaling, cytoskeletal organization, and GBM invasion [30,31], while CASP8 has been implicated in both apoptotic and non-canonical inflammatory signaling [32,33]. Although CXCL12, ESR1, IL4, TNF, and miR-145 were not significantly differentially expressed in the bulk TCGA dataset, these observations are biologically plausible because several of these molecules are predominantly expressed within immune or stromal compartments, are regulated in a context-dependent manner, or, in the case of miR-145, are not measured in conventional mRNA sequencing datasets [34]. Collectively, these findings indicate that the computationally predicted molecular bridge is partially supported by human GBM transcriptomes. It is important to note, however, that the TCGA-GBM cohort lacks documented alcohol exposure history; consequently, this transcriptomic evidence supports the ADAM17-associated molecular network in glioblastoma broadly but cannot directly link these expression changes to EtOH exposure specifically, creating a gap between the EtOH-inclusive network model and the patient-derived validation dataset.

The mechanistic role of ADAM17 was further supported by the computational perturbation analyses. ADAM17 inhibition from the molecular network eliminated the association with glioblastoma despite preservation of the remaining network architecture, indicating that the presence of the surrounding molecular network alone was insufficient to maintain the predicted GBM-associated signaling state (Figure 7). Conversely, retaining ADAM17 as the central hub while removing lateral interactions among the effector molecules produced only a partial GBM association, suggesting that ADAM17 functions as a critical signaling hub whose signaling influence is amplified through cooperative interactions among the broader molecular network (Figure 8). Finally, removal of ADAM17 from the EtOH-centered network eliminated the GBM association despite preservation of the remaining ethanol-associated molecules (Figure 9), further supporting the importance of ADAM17 within the predicted EtOH–GBM signaling axis. Although these perturbation analyses represent computational simulations rather than experimental manipulation, they consistently implicate ADAM17 as the principal organizing node within the predicted network.

Further mechanistic support for the proposed ADAM17-centered network was provided by IPA Upstream Regulator Analysis (URA) of the TCGA-GBM dataset. Although ADAM17 itself was not identified as a predicted upstream regulator, this finding is consistent with the molecular mechanism of ADAM17. URA identifies transcriptional regulators by inferring upstream molecules responsible for the observed downstream gene expression profile [19], whereas ADAM17 functions primarily as a post-translational sheddase rather than a direct regulator of transcription. Consequently, ADAM17 would not be expected to emerge as a conventional upstream regulator despite being an important signaling mediator. It should be noted, however, that this explanation is not the only possible interpretation of ADAM17’s absence from the URA output. An alternative possibility is that the transcriptional changes observed in the TCGA-GBM dataset are simply not associated with ADAM17 activity, and that the predicted activation of TNF, IL-6, ERBB2, EGFR, and TGFA reflects convergent upstream signaling driven by mechanisms independent of ADAM17-mediated shedding. Because URA infers regulator activity from correlated downstream gene expression patterns rather than from direct measurement of proteolytic activity, the presence of activated ADAM17 substrates in this analysis is consistent with, but does not confirm, increased ADAM17 activity. Distinguishing between these possibilities—coordinated ADAM17-driven substrate activation versus independent, ADAM17-unrelated activation of the same downstream mediators—will require direct biochemical assessment of ADAM17 proteolytic activity (e.g., substrate cleavage assays or activity-based probes) in glioblastoma tissue or patient-derived models. Instead, several well-established ADAM17 substrates and downstream signaling mediators, including TNF, IL-6, ERBB2, EGFR, and TGFA, were identified as significantly activated upstream regulators in the TCGA-GBM dataset (Table 1). This association is biologically consistent with increased ADAM17-mediated ectodomain shedding [23], whereby proteolytic release of membrane-bound cytokines, growth factors, and receptors initiate downstream signaling cascades that ultimately drive transcriptional responses detectable by URA. Thus, although URA does not directly identify ADAM17 itself, the activation of its canonical substrate network provides indirect but independent evidence supporting increased ADAM17-associated signaling in human glioblastoma. Importantly, these findings are concordant with both the computationally derived EtOH–ADAM17 network and the differential expression analysis, providing complementary evidence from an independent analytical approach.

The IPA Canonical Pathway Analysis further strengthened this interpretation by demonstrating significant enrichment of multiple pathways closely associated with ADAM17 biology [24]. Among these, the Sheddase Signaling Pathway was significantly enriched [35], providing the most direct patient-derived evidence supporting increased ADAM17 proteolytic activity in GBM. Additional enrichment of EGFR signaling [13], JAK/IL-6 cytokine signaling [36], collagen degradation, ERBB4 signaling [37], neuregulin signaling, and matrix metalloproteinase-related pathways [38] is consistent with the diverse biological functions regulated by ADAM17-mediated substrate shedding [23] (Figure 10). Collectively, these enriched pathways indicate that the molecular consequences of ADAM17 activation extend beyond individual substrates to encompass coordinated signaling programs involved in cell proliferation, inflammation, extracellular matrix remodeling, and tumor invasion, all of which are recognized hallmarks of glioblastoma progression.

The identification of the Acetylcholine Receptor Signaling Pathway as the most significantly enriched canonical pathway within the ADAM17-interactor-filtered TCGA-GBM dataset was unexpected given its lack of an established direct mechanistic link to ADAM17 or EtOH in the context of glioblastoma, and this finding warrants careful biological interpretation. Several considerations support the possibility that this represents a genuine, if indirect, signal rather than a statistical artifact. First, nicotinic acetylcholine receptors, particularly the α7 and α9 subtypes, are expressed by glioblastoma cells, and their activation by choline and nicotine has been shown to promote glioblastoma cell proliferation and survival through downstream signaling cascades, effects that are blocked by nicotinic antagonists or receptor-targeted knockdown [39,40]. Cholinergic receptor activity has also been separately implicated as a modulator of glioblastoma invasion [40], suggesting a broader role for acetylcholine signaling in GBM biology beyond a single pathway. Second, a mechanistic precedent for cholinergic–ADAM17 crosstalk exists in other tissue contexts: in tobacco-smoke-exposed lung epithelial cells, nicotine-derived stimuli have been shown to trigger activation of growth factor signaling through TACE (ADAM17)-mediated transactivation of EGFR, contributing to premalignant hyperproliferative changes [41], and separately, ADAM17 has been shown to promote tobacco-smoke-carcinogen-driven tumorigenesis through shedding of EGFR ligands and the soluble IL-6 receptor [42]. These findings raise the possibility that cholinergic receptor activation and ADAM17-mediated ectodomain shedding converge on shared downstream signaling, such as EGFR transactivation, in a manner that could extend to glioblastoma. Third, an alternative and non-mutually exclusive explanation is that this pathway’s high statistical rank reflects overrepresentation of broadly expressed neuronal and ion-channel-related molecules within the ADAM17-interactor-filtered gene set, rather than a biologically specific cholinergic–ADAM17 interaction; the strongly negative z-score for this pathway, in contrast to the predominantly positive or near-neutral z-scores observed for the sheddase- and EGFR-related pathways, is consistent with either predicted pathway inhibition or a cross-talk-driven compensatory signaling state rather than straightforward pathway activation. Given the absence of direct experimental evidence linking cholinergic receptor signaling to ADAM17 activity in glioblastoma, this finding should be regarded as hypothesis-generating, and future studies directly testing whether nicotinic or muscarinic receptor modulation influences ADAM17 expression or proteolytic activity in GBM models would help clarify whether this represents a true biological signal, an artifact of molecule overrepresentation, or a consequence of convergent downstream signaling cross-talk.

Taken together, the differential expression analysis, Upstream Regulator Analysis (URA), and Canonical Pathway Analysis provide three complementary lines of evidence derived from the TCGA-GBM dataset. Differential expression analysis demonstrates that several components of the proposed network exhibit altered expression patterns in patient tumors, URA identifies predicted upstream regulatory activity involving signaling mediators associated with ADAM17-mediated signaling, and Canonical Pathway Analysis identifies enrichment of biological pathways associated with ADAM17-related functions. The convergence of these complementary analyses strengthens the biological plausibility of the proposed EtOH–ADAM17–GBM signaling axis and illustrates the value of integrating literature-derived mechanistic knowledge with patient-derived transcriptomic data to generate insights into complex disease biology.

**Limitations:** Several limitations of this study should be acknowledged. First, the analyses were computational and hypothesis-generating or correlative in nature and therefore do not establish a causal relationship between ethanol (EtOH) exposure, ADAM17 signaling, and glioblastoma. Experimental studies will be required to determine whether the predicted molecular relationships are biologically active and functionally relevant. Second, the QIAGEN Knowledge Base (QKB) and Ingenuity Pathway Analysis (IPA) rely on curated relationships extracted from the published biomedical literature. Although this knowledge base is extensive and continually updated, it may not capture all relevant molecular interactions and may be influenced by greater representation of well-studied molecules and signaling pathways. Relatedly, the directional filtering approach used to identify EtOH-associated intermediates of ADAM17 signaling does not capture potential feedback relationships in which molecules identified as downstream effectors of ADAM17 might, in turn, influence ADAM17 activity through separate regulatory loops; such bidirectional relationships were outside the scope of the present directional analysis and represent a candidate area for future network-level investigation. Third, the TCGA-GBM analysis represents a secondary analysis of publicly available, previously generated transcriptomic data rather than newly collected patient samples. Therefore, the present findings are constrained by the original cohort design, sample composition, sequencing characteristics, and available clinical annotations. While this approach enables evaluation of molecular patterns in a large patient-derived dataset, additional studies using prospectively collected samples and experimental approaches will be required to confirm the biological mechanisms underlying the proposed EtOH–ADAM17 signaling axis. Fourth, the TCGA-GBM differential expression analysis compared 284 primary GBM tumors against only 5 normal brain tissue samples. This substantial group imbalance limits the statistical power to detect moderate-magnitude expression changes and increases the influence of variability within the small normal-tissue group on differential expression estimates, particularly for genes with smaller effect sizes. While genes meeting the stringent significance thresholds applied in this study (FDR < 0.01, |log2 fold change| ≥ 1) are likely to reflect robust expression differences, this imbalance means that the current analysis may underestimate the true number of differentially expressed network components, particularly among molecules with more modest transcriptional changes. Notably, the limited number of normal brain samples reflects an inherent characteristic of the TCGA-GBM dataset itself rather than a sample-selection decision made in the present study; other TCGA-based analyses have encountered this same constraint and have similarly addressed it through supplementary validation using independent normal-tissue reference sets, such as the Genotype-Tissue Expression (GTEx) project [43]. To address this concern, we performed a supplementary validation analysis using GEPIA3 [21], which incorporates a substantially larger and more balanced normal-tissue comparison (166 GBM vs. 110 GTEx normal brain samples). Five of the six key network molecules evaluated in the original analysis remained significantly differentially expressed in this independent comparison, providing supportive evidence that the original findings are not solely attributable to the limited size of the TCGA normal-tissue group; PRKCD did not reach significance in this analysis and should be interpreted with appropriate caution. Fifth, the TCGA-GBM dataset comprises transcriptomic data from a heterogeneous patient population in which alcohol exposure history was not recorded or controlled. Consequently, the patient-derived analyses provide independent transcriptomic evidence consistent with the predicted ADAM17-associated signaling network but cannot directly evaluate the effects of EtOH exposure, resulting in a disconnect between the EtOH-inclusive computational network and the patient-derived validation data: the TCGA analyses validate the ADAM17-centered portion of the network but not its EtOH-associated component. Future prospective studies incorporating documented alcohol consumption history; for example, cohorts with self-reported or biomarker-confirmed alcohol use linked to tumor transcriptomic or proteomic profiling—will be required to directly test whether EtOH exposure is associated with the predicted molecular changes in ADAM17-associated signaling in glioblastoma patients. Sixth, because TCGA data are derived from bulk RNA sequencing, they do not resolve cell type-specific gene expression and may underestimate the contribution of molecules predominantly expressed in stromal or immune cell populations. In addition, transcriptomic data cannot directly measure ADAM17 proteolytic activity, which is regulated primarily at the post-translational level.

Despite these limitations, the convergence of multiple complementary analyses, including network topology, differential gene expression, Upstream Regulator Analysis, Canonical Pathway Analysis, and custom pathway activation analyses, together with concordant evidence from both a curated literature-derived knowledge base and an independent patient transcriptomic dataset, provides a strong rationale for future experimental investigation of the proposed EtOH–ADAM17–glioblastoma signaling axis.

**Future Directions:** Several directions for future investigation follow directly from the present findings. First, whether EtOH directly modulates ADAM17 expression or proteolytic activity in glioblastoma should be tested experimentally using EtOH-exposed GBM cell lines and in vivo models, alongside evaluation of the effects of ADAM17 inhibition—using established agents such as MEDI3622 or INCB7839—on EtOH-associated tumor progression, invasion, and treatment resistance. Second, the clinical significance of ADAM17 in this cohort could be further characterized by correlating ADAM17 expression with overall and recurrence-free survival in TCGA-GBM patients, applying computational cell-type deconvolution methods such as CIBERSORT or xCell to resolve which cellular populations within the tumor microenvironment contribute to ADAM17 expression, and, where feasible, validating the key network findings using single-cell RNA sequencing (scRNA-seq) data to capture cell type-specific expression patterns not resolved by bulk transcriptomic analysis. Third, because the present TCGA-GBM analysis was constrained by a small normal-tissue control group, validation using independent, more balanced control datasets—such as normal brain tissue samples from the Genotype-Tissue Expression (GTEx) project—would strengthen confidence in the differential expression findings reported here and help clarify whether additional network components show more modest but biologically meaningful expression changes. Fourth, functional validation of the individual bridging molecules identified in this study, particularly CAV1, FLNA, and CASP8, using knockdown or overexpression approaches in EtOH-exposed GBM models, would help establish whether these molecules causally contribute to ADAM17-associated glioblastoma signaling or instead represent correlative markers of the broader network state. Fifth, the unexpected enrichment of the Acetylcholine Receptor Signaling Pathway warrants direct experimental follow-up, including testing whether nicotinic or muscarinic receptor modulation influences ADAM17 expression or proteolytic activity in glioblastoma models, to clarify whether this reflects a genuine cholinergic–ADAM17 signaling link, convergent downstream cross-talk, or an artifact of molecule overrepresentation within the filtered dataset. Sixth, cross-validation of the predicted EtOH–ADAM17–glioblastoma network using orthogonal bioinformatic resources and independent gene expression datasets beyond QKB and TCGA-GBM would help determine whether the computational predictions reported here are robust to the specific knowledge base and cohort used. Finally, the clinical relevance of the proposed signaling axis should be assessed directly in patient cohorts with documented alcohol exposure history, which would allow the EtOH-associated and ADAM17-centered components of the network to be evaluated together rather than separately, as was necessary in the present study. Together, these investigations will be essential for determining whether the computationally predicted, candidate EtOH–ADAM17–glioblastoma molecular axis represents a biologically relevant mechanism with therapeutic implications.

## 4. Materials and Methods

### 4.1. IPA Network Construction

All molecular network analyses were performed using IPA. IPA constructs molecular interaction networks using the QKB, a manually curated repository of molecular relationships derived from the peer-reviewed biomedical literature. Independent molecular networks were generated using ethanol (EtOH) and ADAM17 (TACE) as separate seed molecules. For each analysis, network expansion included both direct and indirect molecular relationships, including activation, inhibition, expression, and binding interactions, using identical IPA settings to ensure comparability between networks. The resulting EtOH- and ADAM17-associated networks were subsequently used for comparative analyses. All the data were obtained from January 2026 to June 2026.

### 4.2. Network Comparison and Identification of Shared Molecules

The EtOH- and ADAM17-associated networks were compared using IPA’s Compare function to identify molecules common to both networks, yielding 97 overlapping molecules. Of these, 10 were categorized as chemical drugs or toxicants and excluded as non-biological intermediates. The directional relationship between EtOH and ADAM17 was evaluated using IPA’s Path Explorer tool, with EtOH set as the origin node and ADAM17 set as the terminal node; only the EtOH-to-ADAM17 directional query was performed. This query returned 53 molecules situated along a biological path connecting EtOH to ADAM17, each linked to EtOH by a colored (orange or blue) edge indicating a confirmed, direction-specific causal effect. The remaining 34 molecules were not returned by this forward-directional query; rather, these molecules were identified as downstream effectors of ADAM17, meaning their documented relationship to ADAM17 runs in the reverse direction (from ADAM17 toward the molecule) rather than from EtOH toward ADAM17, and were therefore excluded as they do not represent EtOH-associated intermediates positioned upstream of ADAM17. Molecules identified in the directional query were subsequently evaluated based on the nature of their connecting edge to ADAM17, as classified within IPA’s network visualization: molecules displaying colored directional edges (orange, predicted activation; blue, predicted inhibition) were considered to represent a continuous, consistent causal path from EtOH through the intermediate to ADAM17, and were retained (n = 25); molecules displaying uncolored (gray) edges specifically to ADAM17, indicating that IPA could not generate a directional prediction for this relationship despite a confirmed colored edge from EtOH, were excluded (n = 28), consistent with IPA’s underlying causal analysis algorithm [19]. This classification is generated directly and reproducibly from IPA’s Path Explorer output; because the retain/exclude decision for each molecule is determined by IPA’s causal prediction algorithm rather than independent researcher interpretation of primary literature, no separate manual curation or inter-rater consensus process was performed at this stage. The remaining 25 molecules were retained as putative EtOH-associated intermediates of ADAM17 signaling and carried forward for all downstream disease association, network integration, computational perturbation, and transcriptomic analyses.

### 4.3. Disease Association and Network Perturbation Analyses

Disease enrichment analyses were performed using the IPA’s Grow- Disease and Functions function. Statistical significance was calculated within IPA using a right-tailed Fisher’s Exact Test, which evaluates whether the overlap between the input molecular set and disease-associated molecules in QKB exceeds that expected by chance.

Disease association analyses were performed for the curated ADAM17-associated molecular set and for the integrated EtOH–ADAM17 network. To evaluate the specificity of the integrated network, a molecular specificity control analysis was performed in which the EtOH-associated bridging molecules were replaced with non-bridging molecules while ADAM17 was retained within the network.

To further evaluate the contribution of ADAM17 to the predicted architecture, three computational perturbation analyses were performed within IPA: (i) simulated ADAM17 inhibition from the complete molecular network while retaining all other network components, (ii) retention of ADAM17 as the sole central hub following removal of lateral connections among the effector molecules, and (iii) simulated removal of ADAM17 from the EtOH-centered network. These analyses were designed to assess the contribution of ADAM17 to the overall network connectivity and its association with glioblastoma.

The ADAM17 inhibition analysis (i) was performed using IPA’s Molecule Activity Predictor (MAP) tool, which allows a selected molecule to be set to a defined activity state (e.g., inhibited or activated) and computes the resulting predicted directional effects on upstream and downstream molecules within the network based on curated causal relationships in the QKB [19]. By contrast, analyses (ii) and (iii) involved direct structural modification of the network, removal of lateral inter-molecular connections in (ii), and deletion of the ADAM17 node itself in (iii). This distinction is important: MAP-based inhibition (i) models ADAM17 in a reduced-activity state while preserving its position and connections within the network, whereas the structural removal used in (ii) and (iii) eliminates specific connections or the node entirely, testing network dependence on ADAM17 through a fundamentally different computational manipulation.

### 4.4. Secondary Analysis of TCGA-GBM Transcriptomic Data

To validate the IPA-derived molecular network in human glioblastoma, differential gene expression data from TCGA-GBM cohort were analyzed. Pre-analyzed datasets were acquired from QIAGEN Omicsoft Land version 2026R2. The dataset comprised RNA sequencing data from 284 primary GBM tumors and 5 normal brain tissue samples generated using the DESeq2 differential expression pipeline (DESeq2 v1.42.1). Sequence reads were aligned to the human reference genome (GRCh38) using the OmicSoft Gene Model V33.

Differentially expressed genes corresponding to molecules identified in the IPA network were evaluated using precomputed log_2_ fold-change and Benjamini–Hochberg false discovery rate (FDR) values provided within the QKB. Genes meeting an FDR threshold of <0.01 and an absolute log_2_ fold-change ≥ 1 were considered significantly differentially expressed. Molecules that did not meet these criteria were retained for descriptive comparison but were not considered significantly altered.

### 4.5. Supplementary Validation Analysis Using GEPIA3

To address the limited number of normal brain tissue samples available in the TCGA-GBM dataset (n = 5), a supplementary validation analysis was performed using GEPIA3 (Gene Expression Profiling Interactive Analysis 3) [21], a web-based tool that integrates RNA sequencing data from the TCGA and Genotype-Tissue Expression (GTEx) projects to enable comparative gene expression analysis using a larger and more balanced normal-tissue reference set. Differential expression between 166 TCGA-GBM tumor samples and 110 GTEx normal brain samples was assessed using the limma method, with significance thresholds of |log2 fold change| ≥ 1 and q ≤ 0.05. This analysis was performed independently of the primary IPA-based TCGA-GBM differential expression analysis described in Section 4.4, and was used solely to evaluate whether the key molecules identified in the original analysis remained differentially expressed when compared against a substantially larger and more balanced normal-tissue reference group.

### 4.6. IPA Upstream Regulator and Canonical Pathway Analyses

The TCGA-GBM differential expression dataset was further analyzed using the IPA Upstream Regulator Analysis (URA) and Core Analysis workflows. URA was used to identify signaling molecules predicted to modulate the observed transcriptomic changes based on known regulator–target relationships within the QKB. Upstream regulators with an absolute activation z-score ≥ 2 and *p* < 0.05 were considered significantly predicted as activated or inhibited, consistent with the activation z-score algorithm described by Krämer et al. [19] and the interpretation convention widely applied in IPA-based studies. Canonical pathway enrichment analysis was performed using the IPA Core Analysis workflow to identify biological pathways significantly changed in the TCGA-GBM differential expression dataset. Predicted activation states were evaluated using IPA activation z-scores together with pathway enrichment *p*-values generated by right-tailed Fisher’s Exact Test [19]. Multiple-comparison correction for the pathway enrichment analysis was performed using the Benjamini–Hochberg method implemented in IPA. This procedure was applied independently of the FDR correction used in the differential expression analysis for identification of DEGs.

### 4.7. Data Availability

The TCGA-GBM transcriptomic dataset is publicly available through The Cancer Genome Atlas (TCGA) Research Network and is accessible through both the NCI Genomic Data Commons (GDC) Data Portal and the QKB [22,23,24,25,26,27,28,29,30,31,32,33,34,35,36,37,38,39,40,41,42,44]. IPA-generated network outputs and curated molecular lists are available from the corresponding author upon reasonable request. No new experimental or sequencing data were generated during this study.

## 5. Conclusions

Using an integrated computational systems biology approach, this study identified a candidate molecular framework connecting EtOH-associated molecular signaling, ADAM17-related pathways, and glioblastoma biology. Analysis of the QIAGEN Knowledge Base identified a shared EtOH–ADAM17 molecular network that was significantly enriched for glioblastoma-associated biology, while independent analysis of the TCGA-GBM patient transcriptomic dataset provided complementary transcriptomic evidence consistent with the predicted network. Differential gene expression analysis demonstrated altered expression of several key network components, whereas Upstream Regulator Analysis and Canonical Pathway Analysis identified predicted regulatory states and biological pathways consistent with established ADAM17 biology. Computational network perturbation analyses further identified ADAM17 as a central organizing node within the predicted signaling network, suggesting that ADAM17-associated signaling may represent an important component of the molecular architecture connecting EtOH-associated pathways with glioblastoma. As these analyses are computational and correlative, they should be interpreted as hypothesis-generating rather than confirmatory of a causal EtOH–ADAM17–GBM relationship.

Collectively, these findings support the hypothesis that ADAM17 represents a candidate molecular mediator of EtOH-associated glioblastoma biology and demonstrate the value of integrating a curated literature-derived knowledge base with independent patient-derived transcriptomic data to generate mechanistic insights into complex disease processes. This integrated systems biology strategy provides a framework for prioritizing experimentally testable molecular targets, including ADAM17, CAV1, and FLNA, for future investigation. Because ADAM17 functions through post-translational proteolytic activity rather than transcriptional regulation, it is pharmacologically accessible through existing classes of metalloprotease and sheddase inhibitors, several of which have already been evaluated in other tumor and inflammatory disease contexts.

If validated by future experimental work, this candidate axis carries several potential translational implications worth noting here. Several classes of ADAM17-targeted agents have already advanced through preclinical and early clinical development, including the antibody-based inhibitor MEDI3622, shown to suppress tumor growth in colorectal cancer xenograft models by targeting ADAM17’s role in shedding tumor-relevant cell-surface proteins and cytokines [45], and the dual ADAM10/17 inhibitor INCB7839 (aderbasib), which was used clinically in combination with trastuzumab to treat HER2-positive metastatic breast cancer, with benefit concentrated in patients expressing the p95 HER2 fragment [46]—an early precedent for biomarker-guided ADAM17-pathway therapy that could inform trial design in GBM. Beyond direct enzymatic inhibition, ADAM17 has also been implicated in immune evasion by glioblastoma-initiating cells (GICs): combined inhibition of ADAM17 and ADAM10 has been shown to increase recognition and killing of GICs by natural killer (NK) cells, suggesting that targeting these metalloproteinases may additionally enhance antitumor immune surveillance in glioblastoma [47]. Regarding stratification, elevated ADAM17 expression has independently been shown in prior studies to increase with glioma grade and correlate with reduced patient survival [15], and separately to be upregulated in temozolomide-resistant glioblastoma cells, with higher expression linked to poorer overall survival in glioma cohorts [16]; together, this prior literature suggests ADAM17 expression as a candidate stratification biomarker worth evaluating for identifying patients who may be more responsive to sheddase-targeted therapy or at greater risk of treatment resistance. Regarding alcohol as a modifiable risk factor, population-level evidence linking alcohol consumption to glioma incidence remains mixed and inconsistent across meta-analyses, with some reporting no net association and others suggesting only modest or region-specific effects [48,49]; this inconsistency is more compatible with a possible role for EtOH in tumor progression or treatment resistance in patients who already have GBM, as proposed here, than in tumor initiation, and supports considering alcohol use as a potentially modifiable factor in patient counseling pending direct prospective evaluation.

Subsequent studies should determine whether EtOH directly modulates ADAM17 expression or proteolytic activity in glioblastoma, evaluate the effects of ADAM17 inhibition in experimental models of EtOH-associated tumor progression, and assess the clinical relevance of the proposed signaling axis in patient cohorts with documented alcohol exposure. Together, these investigations will be essential for determining whether the computationally predicted, candidate EtOH–ADAM17–glioblastoma molecular axis represents a biologically relevant mechanism with therapeutic implications.

## Figures and Tables

**Table 1:** Predicted Activated Upstream Regulators Relevant to ADAM17 Signaling in the TCGA-GBM Dataset. All listed regulators exceeded the significance threshold of |z-score| ≥ 2 and *p* < 0.05, consistent with standard IPA Upstream Regulator Analysis interpretation criteria [19].

Upstream Regulator	z-Score	*p*-Value	Relationship to ADAM17
TNF	8.621	3.2 × 10^−52^	ADAM17 substrate
IL6	6.763	1.31 × 10^−32^	ADAM17 pathway
ERBB2	5.417	1.28 × 10^−25^	EGFR family
EGFR	3.795	3.76 × 10^−9^	Activated by ADAM17 ligand shedding
TGFA	3.376	7.75 × 10^−4^	ADAM17 substrate

## Data Availability

The original contributions presented in this study are included in the article. Further inquiries can be directed to the corresponding authors.

## Supplementary Materials

The following supporting information can be downloaded at: https://www.mdpi.com/article/10.3390/ijms27177837/s1. References [50,51,52,53,54,55,56,57,58,59,60,61,62,63,64,65,66,67,68,69,70,71,72,73,74,75,76,77,78,79,80,81,82,83,84,85,86,87,88,89,90,91,92,93,94,95,96,97,98,99,100,101,102,103,104,105,106,107,108,109,110,111,112,113,114,115,116,117,118,119,120,121,122,123,124,125,126,127,128,129,130,131,132,133,134,135,136,137,138,139,140,141,142,143,144,145,146,147,148,149,150,151,152,153,154,155,156,157,158,159,160,161,162,163,164,165,166,167,168,169,170,171,172,173,174,175,176,177,178,179,180,181,182,183,184,185,186,187,188,189,190,191,192,193,194,195,196,197,198,199,200,201,202,203,204,205,206,207,208,209,210,211,212,213,214,215,216,217,218,219,220,221,222,223,224,225,226,227,228,229,230,231,232,233,234,235,236,237,238,239,240,241,242,243,244,245,246,247,248,249,250,251,252,253,254,255] are cited in the Supplementary Materials.
